# Geniposide ameliorated sepsis-induced acute kidney injury by activating PPARγ

**DOI:** 10.18632/aging.103902

**Published:** 2020-11-10

**Authors:** Jinhong Liu, Ning Zhao, Guiling Shi, Hai Wang

**Affiliations:** 1 Pharmacy Department, Tianjin Baodi Hospital, Baodi Clinical College of Tianjin Medical University, Tianjin 301800, China; 2 Medicine Department, Peking University First Hospital, Beijing 100034, China; 3 Pharmacy Department, Tianjin People's Hospital, Tianjin 300121, China; 4 Pediatrics Department, First Affiliated Hospital of Heilongjiang University of Chinese Medicine, Harbin 150040, China

**Keywords:** geniposide, sepsis, acute kidney injury, PPARγ, inflammation

## Abstract

Acute kidney injury is one of the most common complications that occurs in septic shock. An effective therapeutic intervention is urgently needed. Geniposide has been reported to possess pleiotropic activities against different diseases. However, the effect of geniposide on sepsis-induced kidney injury is unexplored. Our study aims to illustrate the mitigative effects of geniposide on sepsis-induced kidney injury and its relevant mechanisms. Sepsis was induced in mice undergoing cecal ligation and puncture (CLP) surgery. Mice were intraperitoneally injected with geniposide (10, 20 and 40 mg/kg) for treatment. The results showed that geniposide ameliorated kidney injury and dysfunction in CLP-induced septic mice, accompanied by reduction of inflammatory response and oxidative stress. We also found that geniposide significantly reduced vascular permeability and cellular apoptosis of the kidney, with increase of Bcl-2 and decrease of Bax and cleaved caspase-3. Moreover, PPARγ was found to be upregulated with the increasing concentration of geniposide. The protection of geniposide against inflammation and apoptosis was recovered by inhibition of PPARγ. Collectively, these results indicate that geniposide could significantly ameliorate acute kidney injury in CLP-induced septic mice and LPS-stimulated HK-2 cells by activating PPARγ. Geniposide might be a potential drug candidate for sepsis-induced kidney injury.

## INTRODUCTION

Sepsis, caused by dysregulated host response to infection, is deemed as a whole-body inflammatory response, which contributes to extensive tissue damage and multiple organ disorders. It has been the leading cause of death in intensive care units (ICUs) [1]. Acute kidney injury (AKI) is one of the most severe and usual complications that occurs during the progress of sepsis, responsible for more than 50% cases of AKI patients in ICUs [2]. Despite the development of clinical care and intensive care, a specific and effective therapy for AKI is still inadequate. According to the statistics, the incidence rate of AKI is up to 70%, and about 5% of the patients develop acute renal failure. Moreover, the morality of AKI is about 50%, and 15% of survivors still depend on renal replacement therapy (RRT) after discharge, seriously threatening the life of people and troubling family and society [2, 3]. Therefore, excavation of new effective strategies for sepsis-induced AKI is important and urgently required.

Geniposide (GE) is a major active ingredient in the fruits of Chinese herbal medicine *Gardenia jasminoides* Ellis (also named as “Zhizi” in China), which is widely applied for stroke treatment in clinic. GE has been confirmed to partake in various biological and pharmacological activities, including anti-inflammatory, anti-tumor, anti-diabetic, neuroprotective, anti-oxidative, anti-apoptotic and hepatoprotective activities [4–10]. Thus, GE is possibly effective and promising for developing novel drugs. The current evidence has proved that GE has protective effects on sepsis-induced myocardial dysfunction by blocking NLRP3 inflammasome-mediated cardiomyocyte apoptosis and pyroptosis [11]. Besides, GE could directly bound to lipopolysaccharide (LPS) and neutralize it in vitro, and protected sepsis model mice through regulating the cytokine levels [12]. Furthermore, GE could protect renal intrinsic cells, including renal tubular epithelial cells, podocytes and renal collecting duct epithelial cells, from kidney disease [13, 14]. Although these protective functions of GE have been reported, there is no evidence elucidating the effects of GE on sepsis-induced AKI.

On the foundation above, the present study aimed to explore the effects of GE in sepsis-induced AKI, and understand the involved molecular mechanisms.

## RESULTS

### Effects of GE on sepsis-induced kidney injury and dysfunction

**Figure 1:**
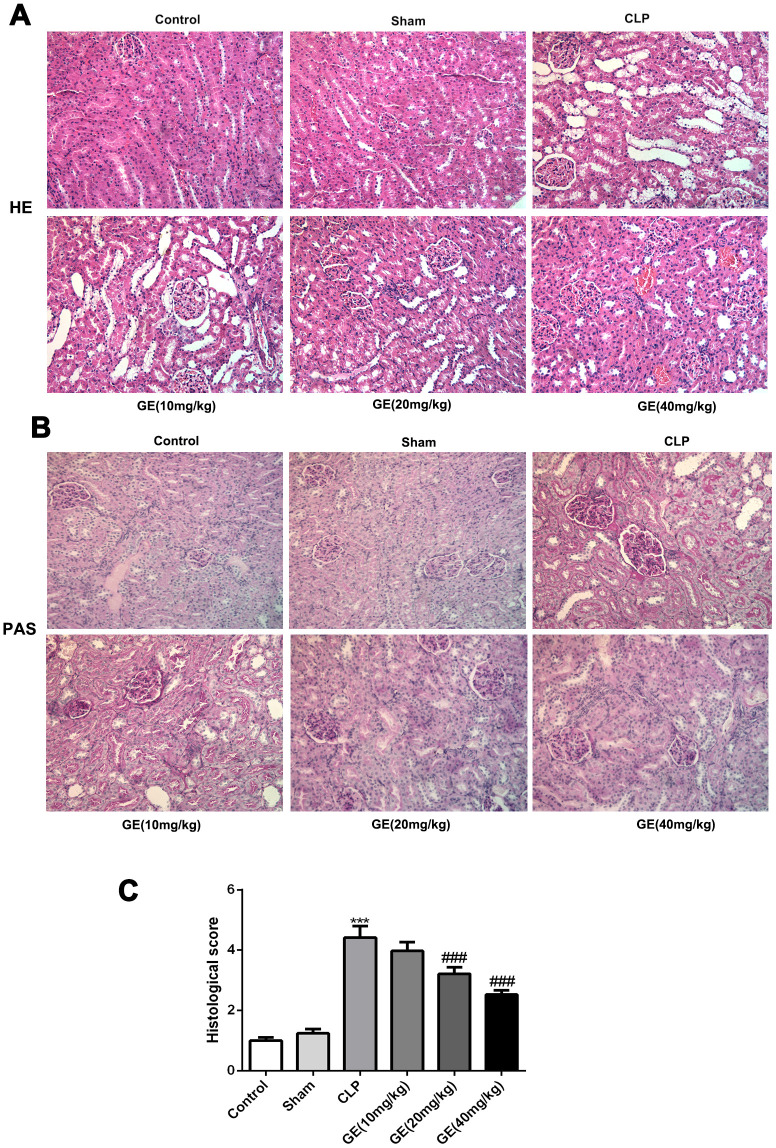
**Effect of GE on sepsis-induced kidney injury.** Sepsis-induced kidney injury model in BALB/c mice was conducted using cecal ligation and puncture (CLP) surgery, and the mice were received GE 1 h after the CLP surgery at different doses (10 mg/kg, 20 mg/kg and 40 mg/kg) three times (once every 6 h). 24 h after the surgery, H&E staining and PAS staining were performed to observe the histopathological changes (**A**, **B**). The histological injury was quantified (**C**). ***p<0.001 vs the sham group. ###p<0.001 CLP group.

The severity of CLP surgery-caused kidney injury was determined by HE and PAS staining. The result showed that the structure of glomerulus was clear and complete, and the kidney tubules were tightly packed in control and sham group. However, in CLP group, glomerulus was shrunken and the kidney tubules exhibited diffuse expansion. Then, GE treatment weakened these histological changes with a dose-dependent way, in agreement with the quantified result (Figure 1A, 1C). Besides, PAS staining showed a severe histological kidney injury as glomerular basement membranes markedly thickened companied with glomerular hypertrophy in CLP group, which was attenuated by GE treatment (Figure 1B).

**Figure 2:**
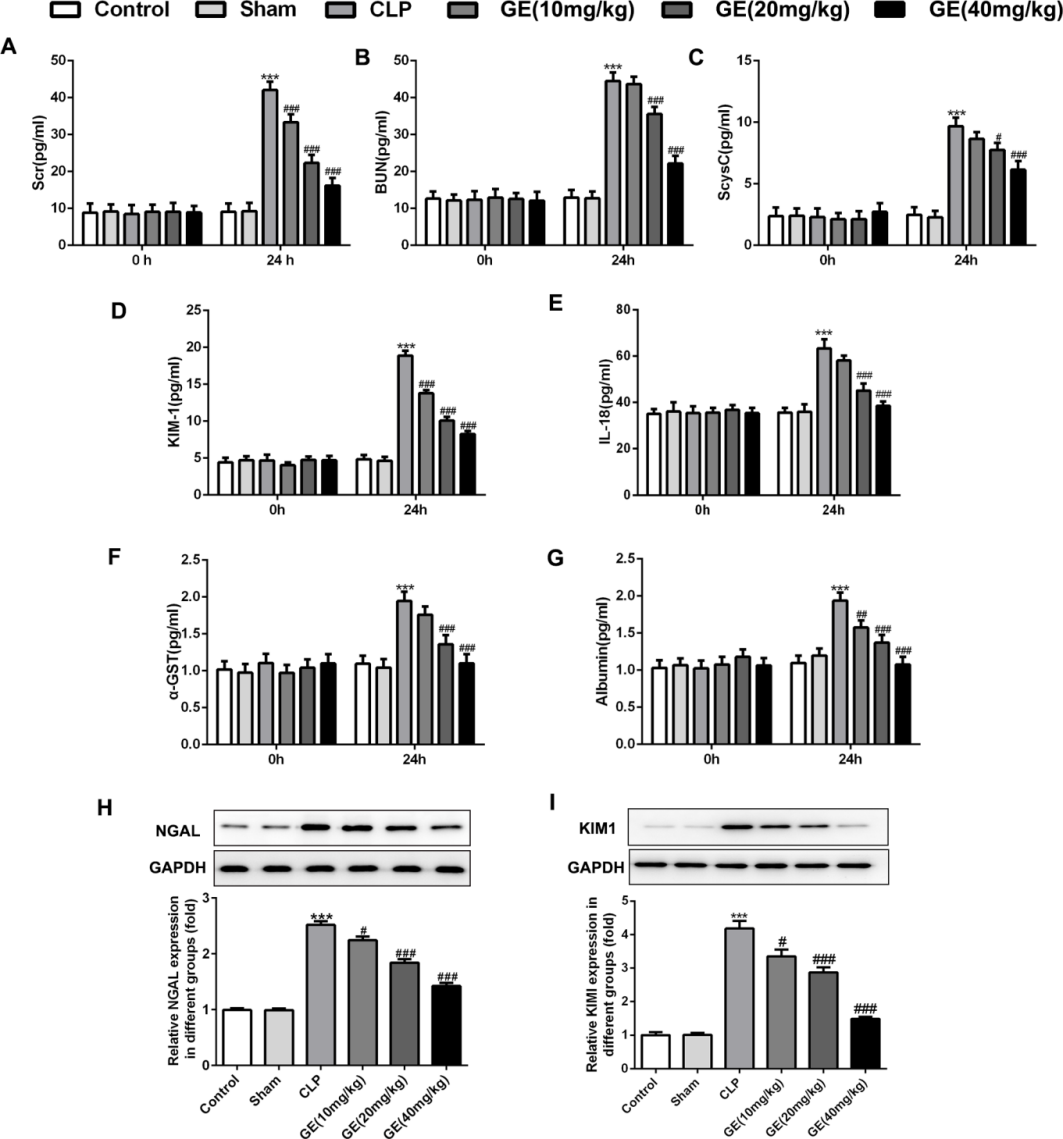
**Effect of GE on sepsis-induced kidney dysfunction.** Levels of serum creatinine (Scr) (**A**), blood urea nitrogen (BUN) (**B**) and serum cystain C (ScysC) (**C**) in serum and α glutathione S transferase (α-GST) (**F**) and albumin (**G**) in urine were measured by an automatic biochemical analyzer. The levels of kidney injury molecule-1 (KIM1) (**D**) and IL-18 (**E**) were determined using their corresponding ELISA kits. The mRNA level and protein expression of NGAL were measured using qRT-PCR and western blot, respectively (**H**, **I**). ***p<0.001 vs the sham group. #, ##, ###p<0.05, 0.01, 0.001 CLP group.

NGAL, KIM1, Scr, BUN, ScysC, IL-18, α-GST and albumin are identified to be markers in acute kidney [15, 16]. As shown in Figure 2A–2G, the levels of KIM1, Scr, BUN, ScysC, IL-18, α-GST and albumin were similar in each group before CLP surgery, respectively. 24 h after surgery, levels of these markers were increased significantly, and an obvious decrease of them were observed in GE group, especially at 20 mg/kg and 40 mg/kg. Besides, protein expressions of NGAL and KIM1 were both upregulated in CLP group, but downregulated after the treatment of GE with a dose-dependent manner (Figure 2H–2I).

### Effect of GE on sepsis-induced inflammatory response and oxidative injury

**Figure 3:**
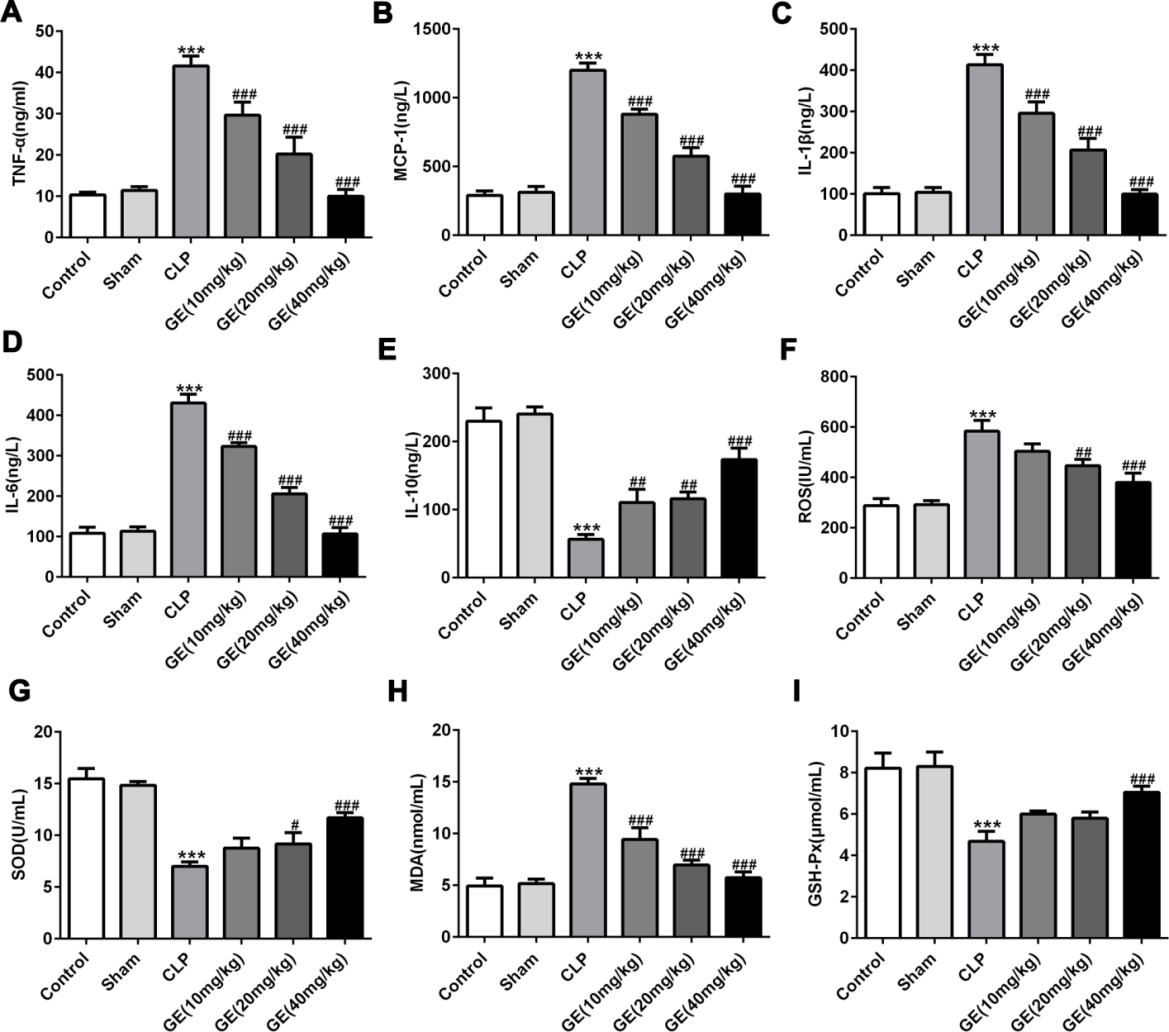
**Effect of GE on sepsis-induced inflammatory response and oxidative injury.** The concentrations of inflammatory cytokines in blood including tumor necrosis factor (TNF)-α, interleukin (IL)-6, IL-10, IL-1β, and monocyte chemotactic protein 1 (MCP-1) in blood were measured using ELISA kit (**A**–**E**). Superoxide dismutase (SOD), malondialdehyde (MDA) and glutathione peroxidase (GSH-px) and reactive oxygen species (ROS) were measured using their corresponding test kits (**F**–**I**). ***p<0.001 vs the sham group. #, ##, ###p<0.05, 0.01, 0.001 CLP group.

Inflammatory cytokines in blood were detected by ELISA assay. The results in Figure 3A–3E displayed that CLP surgery markedly increased the levels of pro-inflammatory mediators such as TNF-α, IL-6, IL-1β, and MCP-1, and decreased the level of anti-inflammatory mediator such as IL-10, while these changes were significantly reversed by introduction of GE. Additionally, the oxidative stress-associated factors were also assessed by corresponding test kits. The results in Figure 3F–3H showed that the levels of ROS, SOD and MDA were higher, and the level of GSH-px was lower in CLP group than those in control group, hinting the severe oxidative stress after CLP surgery. As expected, while GE treatment reversed the effects caused by CLP surgery. These results together suggested that GE could effectively alleviated sepsis-induced inflammatory response and oxidative injury.

### Effect of GE on sepsis-induced vascular permeability

**Figure 4:**
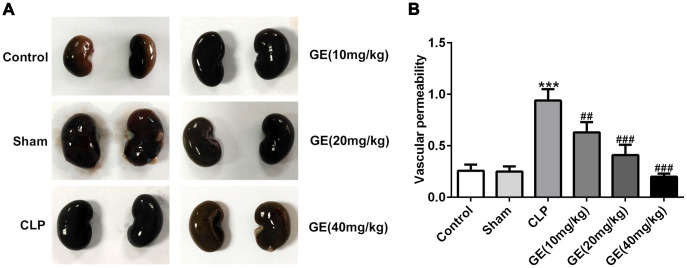
**Effect of GE on sepsis-induced vascular permeability.** Evans blue dye was injected intraperitoneally into the mice, and kidney tissues were collected and observed after 24 h (**A**). The amount of Evans blue dye in the supernatant of kidney tissue was analyzed by detecting absorbance at 620 nm (**B**). ***p<0.001 vs the= sham group. ##, ###p<0.01, 0.001 CLP group.

Changes in vascular permeability are important in the pathogenesis of sepsis-induced organ injury [17]. Therefore, we investigated the changes of kidney vascular permeability using Evans blue dye. The kidney tissues isolated from mice showed that the kidney in CLP group exhibited black, compared with the control tissues, and after the treatment of GE (20 mg/kg and 40 mg/kg), the kidney turned to be nearly normal (Figure 4A). Then the amount of Evans blue dye leakage reflected the degree of kidney vascular permeability. As shown in Figure 4B, CLP significantly increased kidney vascular permeability, which was alleviated by GE. These results suggested that GE could effectively ameliorate sepsis-induced kidney vascular permeability.

### Effect of GE on sepsis-induced kidney tissue apoptosis

**Figure 5:**
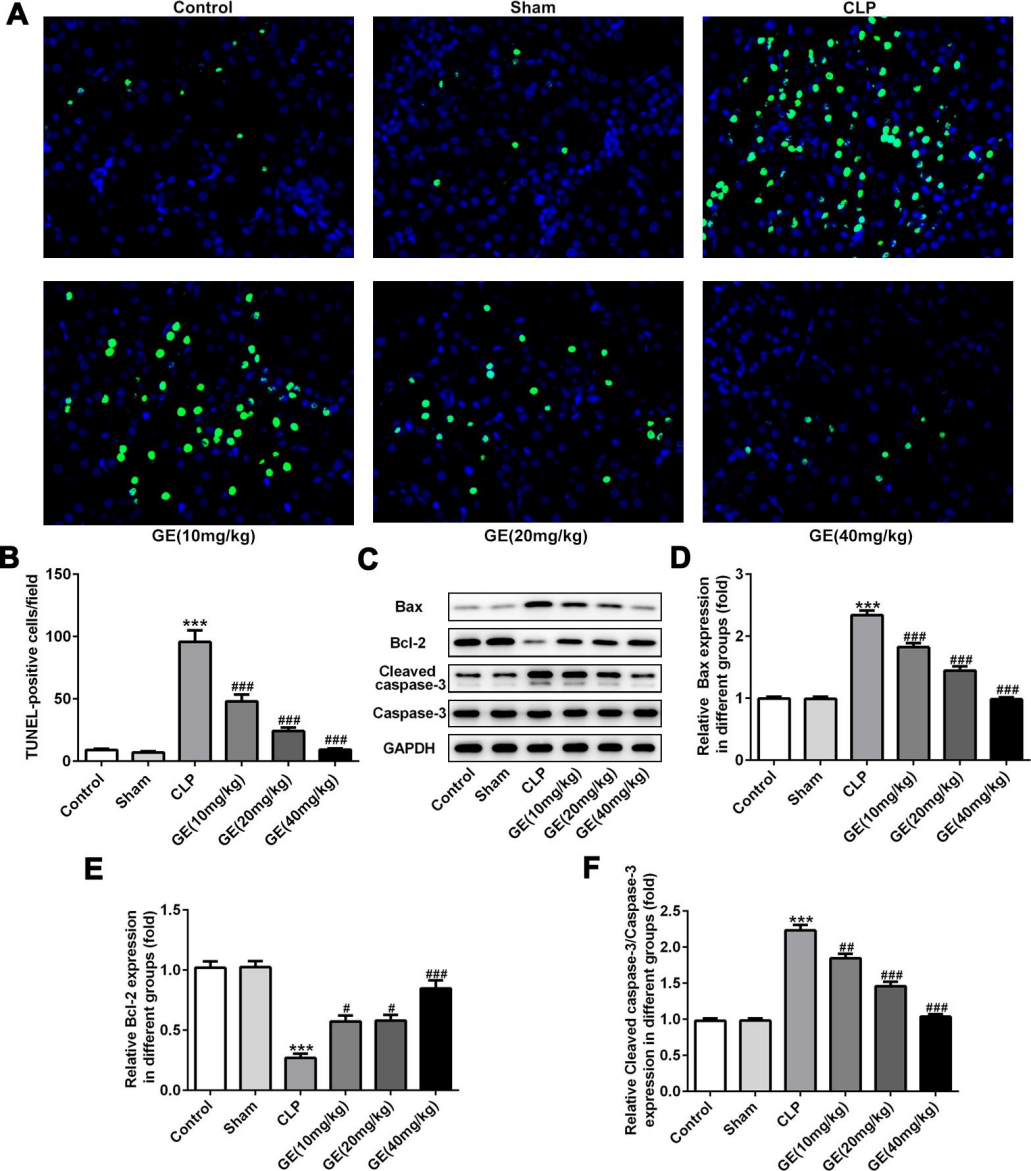
**Effect of GE on sepsis-induced kidney tissue apoptosis.** The apoptosis of cells in kidney was detected using TUNEL assay (**A**). The TUNEL-positive cells were quantified (**B**). The expression levels of apoptosis-related proteins such as Bcl-2, Bax, cleaved caspase 3 and caspase 3 were measured with western blot (**C**–**F**). ***p<0.001 vs the sham group. #, ##, ###p<0.05, 0.01, 0.001 CLP group.

As an important reason for sepsis-induced kidney injury, apoptosis of cells in kidney could result in damage to the septic kidney [3]. The apoptotic cells were detected using TUNEL assay. Compared to the control group, significant promotion of the number of TUNEL-positive cells was observed in CLP group. However, GE obviously declined the number of TUNEL-positive cells at a dose-dependent manner (Figure 5A). Besides, the positive cells were quantified to indicate cellular apoptosis of the kidney tissue (Figure 5B). Consistent with the results of TUNEL assay, the CLP surgery also provoked reduction of Bcl-2 and elevation of Bax and cleaved caspase 3, and the treatment of GE remarkably increased the expression of Bcl-2 and decreased the expression of Bax and cleaved caspase 3(Figure 5C–5F). These results displayed that GE could dramatically reduce apoptotic activity in the cells of the kidney during sepsis.

### Effect of GE on PPARγ expression

**Figure 6:**
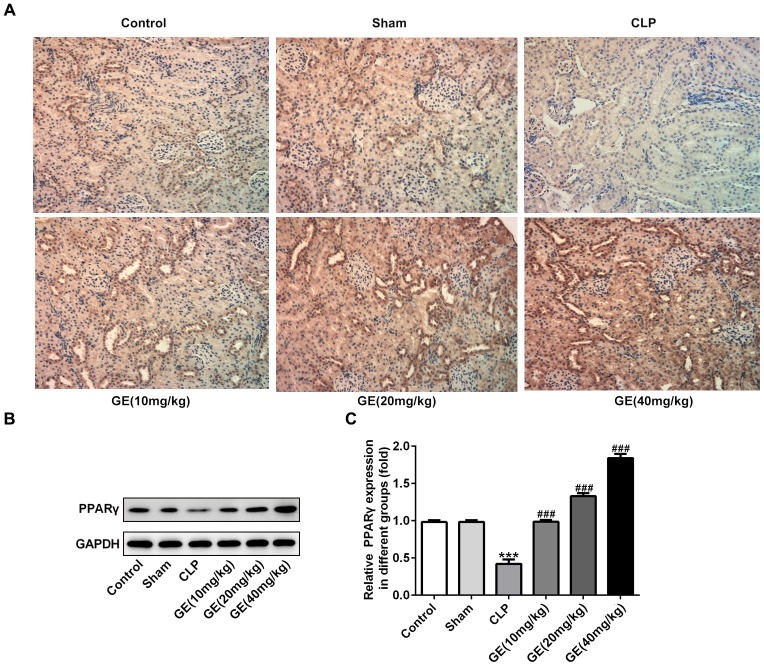
**Effect of GE on PPARγ expression.** The expression of PPARγ in kidney tissue of each group was determined by immunohistochemical analysis (**A**). Besides, western blot was performed to determine the protein expression of PPARγ (**B**, **C**). ***p<0.001 vs the sham group. ###p<0.001 CLP group.

To further understand the mechanism of GE in sepsis-induced kidney injury, we measured the effect of GE on PPARγ signaling using western blot and immune histochemical analysis. In immunohistochemical analysis, the expression of PPARγ was markedly reduced in CLP group in contrast to the control group, which was increased by the GE treatment (Figure 6A). Besides, the western blot assay also exhibited a similar change as the protein expression of PPARγ was distinctly lowered after CLP surgery, and the decrease was alleviated by the treatment of GE (Figure 6B).

### Inhibition of PPARγ abolished the effect of GE

**Figure 7:**
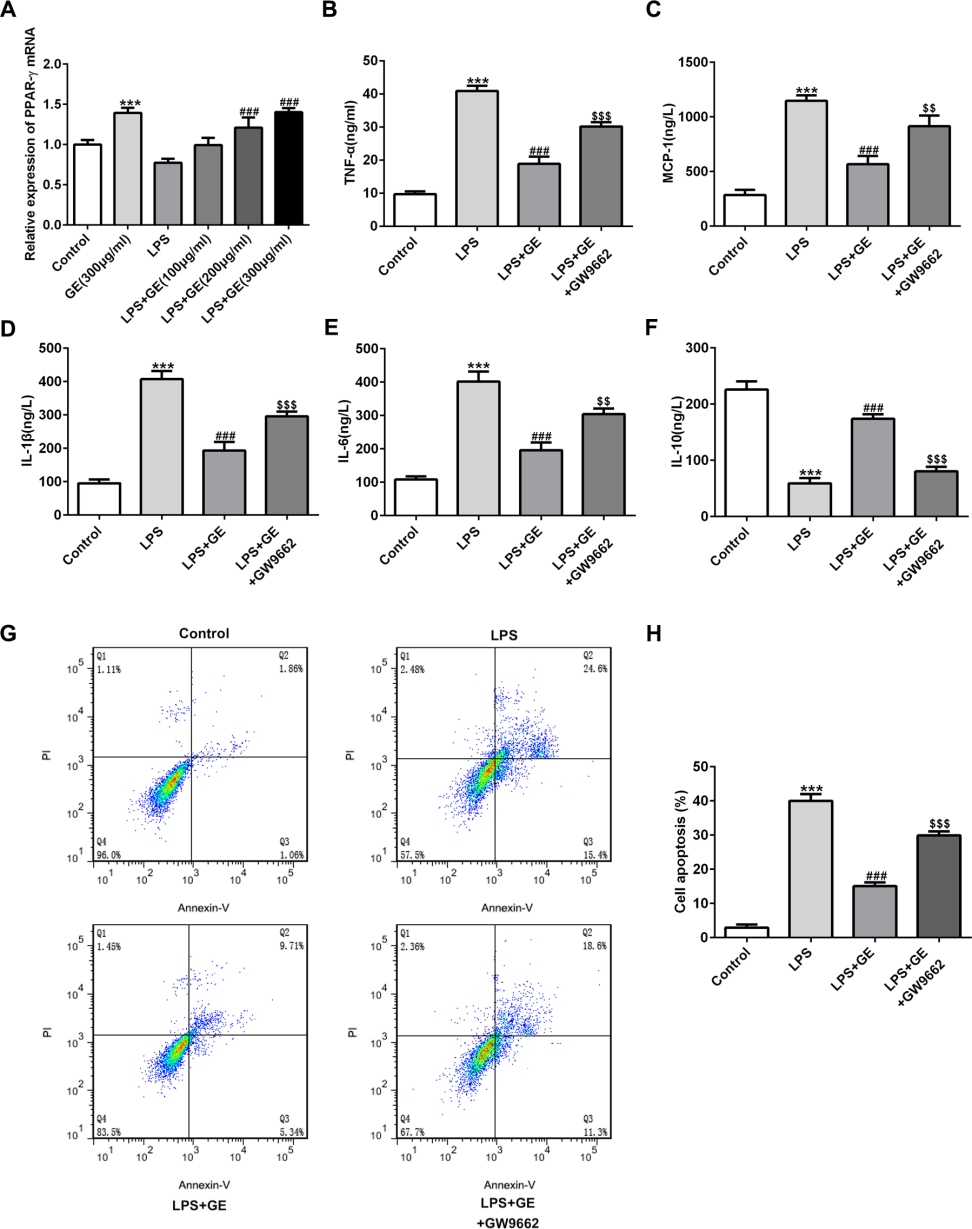
**Inhibition of PPARγ abolished the effect of GE.** LPS-induced HK-2 cells was utilized to simulate sepsis-induced kidney injury. Cells were treated with GE (100 μg/ml, 200 μg/ml, 300 μg/ml), and the mRNA level of PPARγ was detected by qRT-PCR (**A**). GW9662, a PPARγ antagonist, was employed to treat HK-2 cells, and then the concentrations of inflammatory cytokines including TNF-α, IL-6, IL-10, IL-1β, and MCP-1 in each group were measured using ELISA kit (**B**–**F**). Cell apoptotic rate in each group was determined via usage of flow cytometry analysis (**G**, **H**). **, ***p<0.01, 0.001 vs the control group. ##, ###p<0.01, LPS $$, $$$ p<0.01, LPS+GE.

To investigate whether the activation of PPARγ plays an indispensable role in the protective function of GE in sepsis-induced kidney injury, GW9662, a PPARγ antagonist, was introduced into HK-2 cells. Here, LPS-induced HK-2 cells were used to simulate sepsis-induced kidney injury. In LPS-induced HK-2 cells, the mRNA level of PPARγ was decreased, which was improved under GE treatment in a dose-dependent manner (Figure 7A). The detection of inflammatory mediators of each group showed that LPS significantly increased the levels of TNF-α, IL-6, IL-1β, and MCP-1, and decreased the level of IL-10, consistent with those results discovered in mice with sepsis-induced kidney injury. The treatment of GE significantly reversed the expression changes of TNF-α, IL-6, IL-1β, and MCP-1, and IL-10 (Figure 7B–7F). In addition, GE alleviated the elevated cell apoptotic rate induced by LPS (Figure 7G, 7H). However, the suppressive effects of GE on inflammatory response and cell apoptosis were significantly diminished by GW9662, suggesting that the activation of PPARγ was necessary for GE to exert its function, and GE might exert the protective role in kidney injury by activating PPARγ.

## DISCUSSION

Sepsis is a syndrome of acute impairment of function and organ damage, which is associated with long-time adverse outcomes and dependent on the extent of acute injury superimposed on underlying organ reserve [18]. Kidney is one type of susceptible organ in response to sepsis, and acute kidney injury is frequently one complications of sepsis. GE, an active constituent of *Gardenia jasminoides* Ellis, possesses various biological activities such as anti-inflammation, anti-oxidation and anti-apoptosis [4, 5]. Besides, oral administration of GE is distributed rapidly in vivo and the concentration of GE in the kidney is the highest among all the tissues [19]. Moreover, GE develops kidney protection in both streptozotocin-induced diabetic nephropathy and insulin resistance-induced chronic kidney diseases [14, 20]. However, few studies have investigated the protective of GE in sepsis-induced kidney injury. To our knowledge, this was the first study which investigated (a) the effect of GE on sepsis-induced kidney injury and (b) its potential pharmacological modulation mechanism. Our study clearly indicated that GE played a significantly protective role in sepsis-induced kidney injury with a dose-dependent manner. CLP mouse model could reflect many features of human sepsis, thus it is considered as optimal sepsis model and mostly widely used. Herein, we adopted CLP surgery to conduct sepsis model. GE was validated to alleviate kidney histopathological changes and kidney dysfunction, as well as reduce the oxidative stress and excessive pro-inflammatory cytokines-caused inflammatory response. Besides, GE also decreased kidney vascular permeability and apoptotic cells that were induced after CLP surgery. Mechanically, expression of PPARγ was significantly decreased after CLP surgery, and GE treatment alleviated the reduction. Using HK-2 cells as an *in vitro* model, GE was also affirmed to suppress inflammatory response and cell apoptosis triggered by LPS. However, the inhibition of PPARγ dramatically diminished the anti-inflammatory and anti-apoptotic effects of GE. Therefore, from the results above, it can be concluded that GE has significantly inhibitive impacts on inflammatory response, cell apoptosis, and vascular permeability in sepsis-induced kidney injury through activating PPARγ, and that GE might be a potential drug candidate for sepsis-induced kidney injury.

The essence of sepsis is systemic inflammatory reactions [21]. In sepsis, the uncontrolled inflammatory response is one of the main factors causing multiple organ dysfunction syndrome. The inflammatory cytokines such as TNF-α and IL-1β are of diagnostic and predictive values, and mice deficient in IL-1β was partially protected against damage induced by CLP surgery [22]. In our finding, it was displayed that the pro-inflammatory cytokines such as TNF-α and IL-1β were significantly lessened and the anti-inflammatory cytokine IL-10 was obviously increased after GE treatment in kidney injury *in vivo* and *in vitro*, suggesting that GE had inhibitive influence on uncontrolled inflammatory response in sepsis-induced kidney injury. Additionally, aberrant inflammatory response could destroy the balance of oxidant/anti-oxidant system, thereby producing oxidative stress injury. Meanwhile, the excessive production of cellular ROS in turn aggravates inflammatory reaction by activating key inflammatory signaling pathways such as NF-κB pathway [23, 24]. The results in our study presented that the excessive production of ROS induced by sepsis was attenuated by GE treatment. The downregulated level of SOD and the upregulated levels of MDA and GSH-px induced by sepsis were also weakened after GE treatment, indicating that GE could alleviate sepsis-induced oxidative stress. Moreover, the increased vascular permeability is one hallmark of the sepsis-caused systemic inflammation. After 8 and 24 h of CLP surgery, the vascular permeability increases in all organs of mice [25]. In agreement with the previous reports, our findings discovered that CLP surgery markedly increased vascular permeability of kidney, however GE treatment decreased the vascular permeability in the kidney after 24 h, hinting that GE could mediate vascular permeability during systemic inflammation. Therefore, the results above in our study suggested that GE had potent suppressive effects on inflammatory response, oxidative stress and vascular permeability to protect kidney from sepsis.

Compelling evidence has demonstrated that cell apoptosis, especially apoptosis of renal tubular cells, is involved in the pathogenesis and progression of sepsis-induced kidney injury [26]. The apoptosis of renal tubular epithelial cells is found in the patient’s renal biopsy specimen, in accordance with those of animal septic models where apoptosis emerged as a key factor in the pathogenesis of kidney failure [27, 28]. Also, in CLP surgery-induced sepsis in mouse model, more apoptotic cells in kidney tissues were viewed than the control tissues, and the protective role of baicalin against acute kidney injury in pediatric sepsis might be due to its inhibition on renal cell apoptosis [29]. In the present study, a higher proportion of apoptotic cells in kidney tissues was also observed after CLP surgery *in vivo* as well as in HK-2 cells triggered by LPS *in vitro*. However, the treatment of GE exhibited an obvious inhibitive effect on cell apoptosis. Bcl-2 family proteins are key regulators of apoptosis. The anti-apoptotic protein Bcl-2 and pro-apoptotic protein Bax are two important proteins of this family, which determine the activation of caspase-3 and the severity of apoptosis [21, 30]. The present study disclosed that the expression of Bcl-2 was decreased and the expressions of Bax and cleaved caspase-3 were increased after CLP surgery, which were markedly abrogated by the treatment of GE. These findings demonstrated that GE possessed potent anti-apoptotic activity against sepsis-induced kidney injury.

PPARγ is a member of the nuclear receptor family, belonging to the steroid hormone nuclear receptor family. It has been demonstrated that activation of PPARγ has great effects on modulating inflammatory, immune, fibrotic and proliferative pathway, which are mediated through renal parenchymal and infiltrating cells. When PPARγ is activated, various target genes expressing PPARγ response elements are influenced. For example, activated PPARγ inhibited the expression of pro-inflammatory mediators by inflammatory cells [31]. PPARγ agonist significantly attenuated renal injury by inhibiting oxidative stress and apoptosis in mice through activation of UCP1 [32]. In addition, PPARγ has been proved to hamper inflammation response in sepsis [33]. The previous studies have been reported that in vivo treatment with PPARγ ligands inhibit neutrophil recruitment in major organs of rats with polymicrobial sepsis [34]. The activation of PPARγ reduces sepsis-induced lung injury and inflammatory cell infiltration into intestinal tissues, and also prevents sepsis-induced myocardial dysfunction through reducing pro-inflammatory cytokines, apoptosis and necroptosis [35, 36]. It is interesting that GE has been reported to enhance the phosphorylation of PPARγ and accelerate the release of phosphorylated FoxO1 from nuclear fraction to the cytosol [37]. FoxO1, as a main nucleus factor that regulates transcriptions of genes related to cell metabolism, can bind to the PPARγ promoter region to regulate PPARγ expression and regulate cell cycle distribution and apoptosis of tubular epithelial cells of rat kidney [38], indicating that GE might alleviate kidney injury through regulating PPARγ/FoxO1. Thus, the activation of PPARγ was considered as an optional approach for ameliorating organ damage by inhibiting inflammation and apoptosis, which might be the main approach for the protective role of GE in sepsis-induced AKI in this study. In the present work, the expression of PPARγ in kidney tissues or HK-2 cells was promoted due to the treatment of GE, meaning that GE could improve the expression of PPARγ. However, whether GE generates its function through PPARγ still needs exploration. GW9662, a PPARγ antagonist was employed in HK-2 cells, along with GE treatment to study the role of PPARγ in the protective effect of GE. It could be seen that pre-treatment of GW9662 significantly reversed the protective effects of GE against inflammatory response and cell apoptosis triggered by LPS, indicating that the effects of GE were mediated through activation of PPARγ. GE might develop its protective role in sepsis-induced kidney injury through activation of PPARγ.

## CONCLUSIONS

Taken together, the results of this study demonstrated that kidney dysfunction, inflammatory response, oxidative stress, vascular permeability and cell apoptosis were inhibited by the treatment of GE in sepsis-induced kidney injury. Furthermore, the therapeutic effects of GE on sepsis model *in vivo* and *in vitro* may act by activating PPARγ. Accordingly, GE might be a promising drug candidates for sepsis-induced kidney injury.

## MATERIALS AND METHODS

### Experimental animals and experimental design

All BALB/c mice (male; 25-30 g) were obtained from Vital River Laboratories Co., Ltd. (Beijing, China) and kept in a pathogen-free room with standard laboratory diet and water ad libitum, with stable room temperature (22-24 °C) and 12 h light/dark cycle. All animal experiments were performed following Nursing and Use Guidance for Animal Experiment Operation of National Institutes Health.

Mice were anesthetized by intraperitoneal injection of ketamine (100 mg/kg) and xylazine (10 mg/kg). Sepsis was induced in mice undergoing cecal ligation and puncture (CLP) surgery as previously described [39]. Briefly, the abdominal area was shaved and disinfected, and a midline vertical incision of 2 to 3 cm was made in the abdomen. The cecum was then ligated from the top and punctured twice by piercing the cecum with an 18-gauge needle. After a small amount of stool was squeezed from the puncture hole, the cecum was reinserted afterwards into the abdominal cavity and the incision was sutured. Pre-warmed saline was then administrated intraperitoneally for resuscitation. The sham-operated mice underwent CLP surgery except cecal ligation and perforation. To explore the protective effect of GE, GE groups received an intraperitoneal injection of GE (NO. #SML0153; Sigma-Aldrich, St. Louis, MO, USA) 1 h after the CLP surgery at different doses (10 mg/kg, 20 mg/kg and 40 mg/kg) three times (once every 6 h). 24 h after the surgery, mice were euthanized, and blood samples and kidney tissues were gathered for further analysis.

### Analysis of inflammatory cytokines and oxidative stress-associated factors

The concentrations of inflammatory cytokines including tumor necrosis factor (TNF)-α, interleukin (IL)-6, IL-10, IL-1β, and monocyte chemotactic protein 1 (MCP-1) in blood were measured using corresponding enzyme-linked immunosorbent assay (ELISA) kits (R&D System, Minneapolis, MN, USA). Superoxide dismutase (SOD), malondialdehyde (MDA), and glutathione peroxidase (GSH-px) and reactive oxygen species (ROS) were measured using their corresponding test kits (Nanjing Jiancheng Bioengineering Institute, Nanjing, China).

### Real-time quantitative PCR analysis

Total RNA was extracted from kidney tissues of different groups using TRIzol reagent, and then reversely transcribed into cDNA using PrimeScript Reagent Kit (Takara, Dalian, China). Subsequently, Real-time PCR was performed using the SYBR Mixture (Takara) to determine the gene levels. GAPDH was utilized as an internal control, and the quantification of relative transcript levels was assessed with the comparative threshold (Ct) method.

### Western blot

Total protein was extracted from kidney tissues of different groups using RIPA cell lysis buffer, and then quantified using a BCA kit. The same amount of protein was separated by 10% sodium dodecyl sulfate-polyacrylamide gel electrophoresis (SDS-PAGE) and transferred onto polyvinylidene difluoride (PVDF) membranes. Subsequently, the membranes were blocked with 5% nonfat milk and next incubated with primary antibodies at 4 °C overnight, followed by rinse with PBS for three times and incubation with anti-rabbit and anti-mouse horseradish peroxidase-conjugated secondary antibodies. The bands were developed using enhanced chemiluminescence reagents (Pierce, Rockford, IL, USA).

### Determination of kidney function

Blood samples were made by centrifugation at 3000 rpm for 10 min, and then levels of blood urea nitrogen (BUN), serum creatinine (Scr) and serum cystain C (ScysC) in serum and α glutathione S transferase (α-GST) and albumin in urine were measured by an automatic biochemical analyzer (AU400, Olympus, Japan). The levels of kidney injury molecule 1 (KIM1) and IL-18 were determined using their corresponding ELISA kits (R&D System). The expression of neutrophil gelatinase-associated lipocalin (NGAL) and KIM1 was determined using western blot.

### Histopathological examination

Kidney tissue samples were collected 24 h after surgery, fixed in 10% paraformaldehyde, embedded in paraffin, and cut into sections with 4 μm-thickness. The sections were then stained with hematoxylin and eosin (H&E). Additionally, the sections were stained with periodic acid-schiff (PAS). The histological changes of kidney were observed under a light microscope (Olympus IX71, Tokyo, Japan) at 200X magnification.

### TUNEL analysis

The apoptosis of kidney tissues was measured using TUNEL Assay Kit (KeyGEN BioTECH, Jiangsu, China) as the manufacturer instructed. After deparaffinization and hydration, the sections were washed with PBS, and treated with proteinase K working solution. After that, sections were cultivated with TUNEL reaction mixture at 37 °C for 60 min, followed by incubation with DAPI for 10 min. The results were observed through the fluorescence microscope (Olympus, Japan).

### Evans blue staining

To study vascular leakage from kidney tissues, Evans blue dye (Sigma-Aldrich, St. Louis, MO, USA) was injected intraperitoneally into the mice 30 minutes before sacrifice, and kidney tissues were collected and viewed. Then, Kidney tissues were weighed and homogenized in 1 ml formamide and incubated at 55 °C for 18 h. After centrifugation, the supernatant was collected, and the amount of Evans blue dye in the supernatant was analyzed by detecting the absorbance at 620 nm and calculated according to a standard curve of Evans blue dye.

### Immunohistochemical analysis

Kidney tissues were fixed with paraformaldehyde, embedded in paraffin, and cut into sections for immunostaining with antibody against PPARγ. Subsequently, sections were deparaffinized using a graded series of ethanol, and stained using primary antibody against PPARγ. After washing with PBS, sections were incubated with EnVision+/HRP/Rb (DAKO, Glostrup, Denmark) for 30 min at room temperature. The staining was visualized using 3, 3’-diaminobenzidine (DAB) substrate and then counterstained with hematoxylin for 30 s. All sections were photographed using an Olympus BH2 microscope (Olympus).

### Cell culture and treatment

HK-2 cells were obtained from China Center for Type Culture Collection (CCTCC, Wuhan, China) and cultured in RPMI 1640 medium supplemented with 10% fetal bovine serum (FBS) at 37 °C in a humidified atmosphere with 5% CO_2_.

For experimental study, HK-2 cells were pretreated with GE (300 μg/ml) for 1 h, or 10 μM GW9662 (a PPARγ antagonist) for 30 min before GE incubation (100, 200 and 300 μg/ml) and stimulated with LPS (1 μg/ml) for 24 h.

### Flow cytometry

For apoptotic rate analysis of LPS-induced HK-2 cells, flow cytometry was performed using an Annexin-V-FITC Apoptotic Detection kit (KeyGEN) as guided by the manufacturer. Briefly, after treatment for 24 h, cells were rinsed with PBS and stained with PI/FITC-Annexin V in the presence of RNase A (Sigma-Aldrich). Next, cells were grown for 30 min at room temperature in the dark. A FACScan flow cytometer (BD Biosciences, San Jose, CA, USA) was employed to detect apoptosis within 1 h.

### Statistical analysis

All data were analyzed using Graphpad 4.0 software (Graphpad software, Inc., CA, USA) and SPSS 18.0 software (SPSS Inc., Chicago, IL, USA) and expressed as mean ± standard deviation (SD) from at least three independent experiments. Differences between groups were examined for statistical significance by variance analysis (ANOVA). A P value<0.05 was accepted as statistically significant.
